# Integrated analysis of microRNAs, transcription factors and target genes expression discloses a specific molecular architecture of hyperdiploid multiple myeloma

**DOI:** 10.18632/oncotarget.4302

**Published:** 2015-05-27

**Authors:** Maria Teresa Di Martino, Pietro Hiram Guzzi, Daniele Caracciolo, Luca Agnelli, Antonino Neri, Brian A. Walker, Gareth J. Morgan, Mario Cannataro, Pierfrancesco Tassone, Pierosandro Tagliaferri

**Affiliations:** ^1^ Department of Experimental and Clinical Medicine, Magna Graecia University, Salvatore Venuta University Campus, Catanzaro, Italy; ^2^ Department of Surgical and Medical Sciences, University of Catanzaro, Catanzaro, Italy; ^3^ Department of Clinical Sciences and Community Health, University of Milan, Hematology1 CTMO, Foundation IRCCS Ca' Granda Ospedale Maggiore Policlinico, Milano, Italy; ^4^ Centre for Molecular Pathology, The Royal Marsden Hospital, London, UK; ^5^ Myeloma Institute for Research and Therapy, University of Arkansas for Medical Sciences, Little Rock, AR, USA; ^6^ Sbarro Institute for Cancer Research and Molecular Medicine, Center for Biotechnology, College of Science and Technology, Temple University, Philadelphia, PA, USA

**Keywords:** integromics, microRNA, miRNA, transcription factors, multiple myeloma, hyperdiploid myeloma

## Abstract

Multiple Myeloma (MM) is a malignancy characterized by the hyperdiploid (HD-MM) and the non-hyperdiploid (nHD-MM) subtypes. To shed light within the molecular architecture of these subtypes, we used a novel integromics approach. By annotated MM patient mRNA/microRNA (miRNA) datasets, we investigated mRNAs and miRNAs profiles with relation to changes in transcriptional regulators expression. We found that HD-MM displays specific gene and miRNA expression profiles, involving the Signal Transducer and Activator of Transcription (STAT)3 pathway as well as the Transforming Growth Factor–beta (TGFβ) and the transcription regulator Nuclear Protein-1 (NUPR1). Our data define specific molecular features of HD-MM that may translate in the identification of novel relevant druggable targets.

## INTRODUCTION

Multiple myeloma (MM) is a lethal disease of antibody-secreting bone marrow plasma cells (PCs) that accounts for 10% of all hematological neoplasias with worldwide increasing incidence. MM is characterized by a wide clinical spectrum ranging from the pre-malignant condition called monoclonal gammopathy of undetermined significance (MGUS), to smoldering MM, truly overt and symptomatic MM, and finally extra-medullary MM/plasma cell leukemia (PCL). Despite the recent remarkable improvement in the treatment and patient care, the availability of novel therapeutic strategies and investigational platforms, MM remains an incurable disease [1-11]. In the last decade, important advances in molecular cytogenetics and global genomic studies brought about a significant advancement in the comprehension of MM pathogenesis [12-14]. Nearly half of MM tumors are defined as hyperdiploid (HD-MM) associated with copy number alterations, such as trisomies of odd chromosomes (3,5,7,9,11,15,19,21). The remaining tumors are referred as non-hyperdiploid (nHD-MM) and are frequently associated with constitutive activation of genes, such as *CCND1* (11q13), *CCND3* (6p21), *MAF* (16q23), *MAFB* (20q11), or *FGFR3/MMSET* (4p16.3), as a result of chromosomal translocations involving the immunoglobulin heavy chain locus (IgH) on chromosome 14q32. Even though the genetic characterization of HD-MM and nHD-MM is well established at both mRNA or miRNA levels [15-19], there is still scope for an integrated deep analysis of whole molecular profiling data to disclose transcriptional networks of HD-MM. Li et al [20] have in fact hypothesized that chromosome alterations imprint the gene expression by dosage effect and developed a method for MM classification with a deeply comprehension of the disease biology and prognosis of HD-MM and nHD-MM subtypes, reinforcing the rationale of our investigation.

In the last decade, the role of miRNAs as post-transcriptional regulators has been widely investigated. Deregulation of their expression has been associated with several diseases including cancer. Nonetheless, the elucidation of the mechanisms underlying miRNA involvement in MM pathogenesis is still an unmet goal. Our experimental strategy relies on novel integrative genomics approaches, which integrate data from different genomic levels (mRNA, miRNA, transcription factors, genetic aberrations, methylomics and others) with clinical data, providing a comprehensive view of underlined biology [21-23]. Existing approaches span from the application of classical fold-change analysis of miRNA expression data to the application of complex models of integration of miRNA and mRNA expression data into single networks. However, these latter strategies may suffer from several drawbacks: (*i*) the existence of many false positive miRNA targets, (*ii*) the lack of comprehension of downstream mechanisms, and (*iii*) the presence of feedback and feed-forward loops among miRNAs and genes. Recent findings clearly support the notion that interconnecting miRNAs with transcription factor (TF) genes and other upstream regulators (URs) may be of major help for the elucidation of oncogenetic and progression events [24]: consequently, several novel approaches have been devoted to the investigation of relations among miRNAs, expression of target genes and transcription factors [25]. To this aim, traditional gene-set-enrichment tools [26] have been partially substituted by more sophisticated models based on a system-level or semantic-based analysis [27, 28], and data interpretation by the integration with prior biological knowledge, into the workflow of analysis, e.g. the use of causal networks (i.e. networks that explicitate the causal relationships) [29, 30]. Based on the ability to retrieve the direction of interactions among genes (including mutual feed-forward loop), causal analysis is more efficient than classic enrichment methods. To date, different approaches have been described for casual analysis [21]: herein, we selected those that have been implemented into the Ingenuity Pathway Analysis^®^ (IPA) platform [31]. Specifically, we performed our analysis starting from the hypothesis that trisomies associated with HD-MM may extensively affect miRNAs, transcription regulators (TRs) and target gene expression in this subset of patients. To this aim, we took advantage of IPA analysis and paired miRNA and gene expression data from a recently published dataset [16]. Our approach has been based on a novel 3-step strategy: (*i*) identification of differentially expressed mRNA/miRNAs, (*ii*) identification of regulatory networks in which these genes were involved, by using prior-biological knowledge stored on IPA, and (*iii*) overlapping of miRNA regulatory class with regulatory network data. The final goal is the identification of reliable upstream biological causes and downstream effects, in order to depict the main functional features embedded in the HD-MM genomic architecture.

## RESULTS

### Differential expression analysis

**Figure 1:**
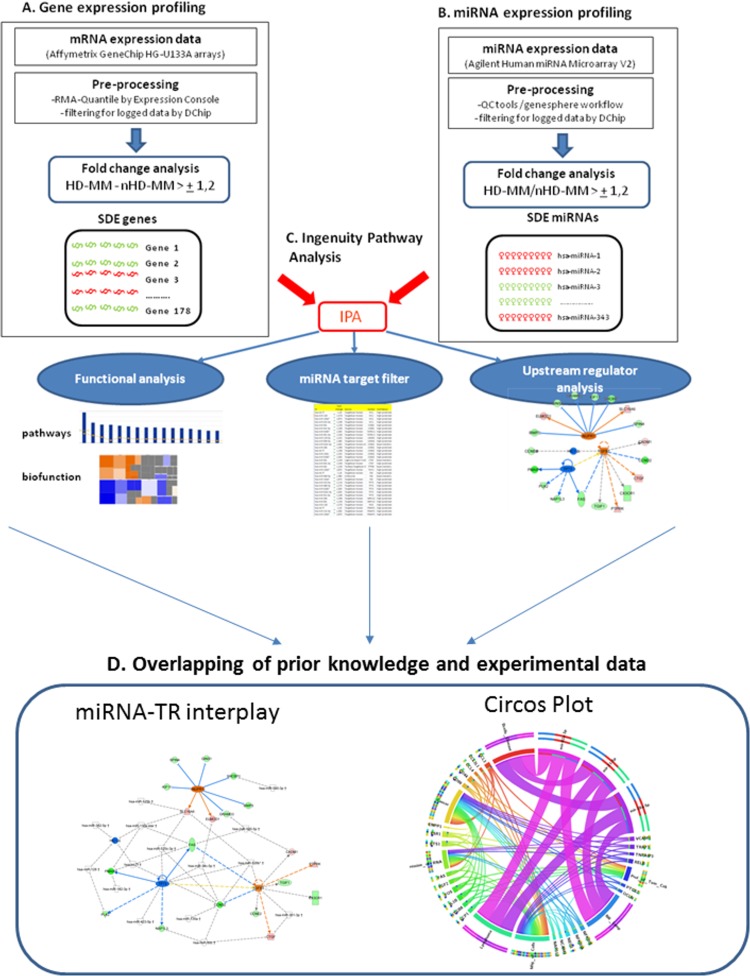
Overview of the workflow used in the analytical model **A.**-**B.** Microarray available data-sets published by Wu et al. were the basic material for all analysis. After the initial preprocessing conducted by Affymetrix proprietary software, we filtered data using DChip. Supervised analysis identified significant differentially expressed (SDE) mRNA and miRNA by comparing two annotated groups hyperdyploids (HD-MM) *versus* non hyperdyploids (nHD-MM) multiple myeloma obtaining two different SDE lists: i) SDE-genes and ii) SDE-miRNAs. **C.** IPA software was used to perform: i) functional analysis (canonical pathways and bio-functions), carried out by SDE-genes and miRNAs to identify biological events related to the two MM subtypes; ii) miRNA target filter, performed by the SDE-miRNAs to select experimental or high confidence predicted target annotated in IPA base; iii) upstream regulator analysis (URA), integrated gene expression data into IPA software to identify URs related to the identified transcription events. **D.** Overlapping of prior knowledge inferred by IPA with experimental data (transcriptional and post-transcriptional) to identify miRNA-transcription regulators interplay and Circos Plot representation to visualize miRNA-gene anti-correlations and inference of the biological behavior in the MM disease.

We analyzed the paired mRNA/miRNA microarray data included in a public available dataset of MM patients at diagnosis from a well-characterized clinical trial [16], aimed at elucidating the UR mRNA-miRNA circuitry depicted in HD-MM *versus* nHD-MM. The workflow of data processing and analysis is outlined in Figure 1. After pre-processing of raw data using Affymetrix tools, we used dChip software to filter possible outlier genes and miRNAs and to compare the two MM subgroups.

**Figure 2:**
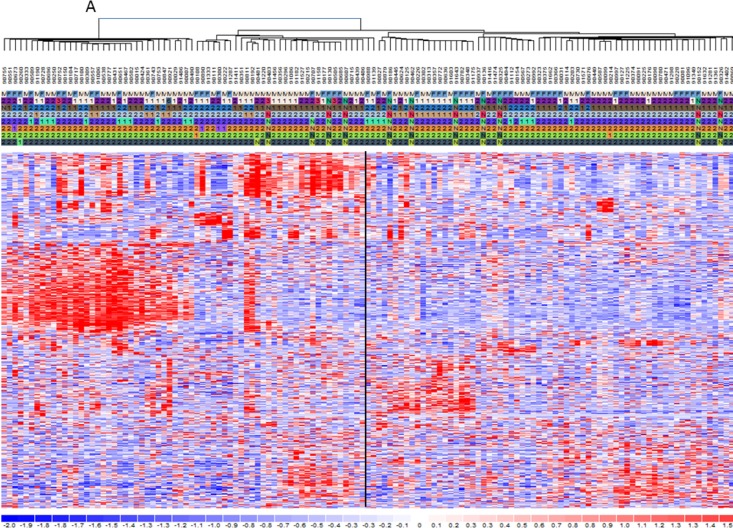

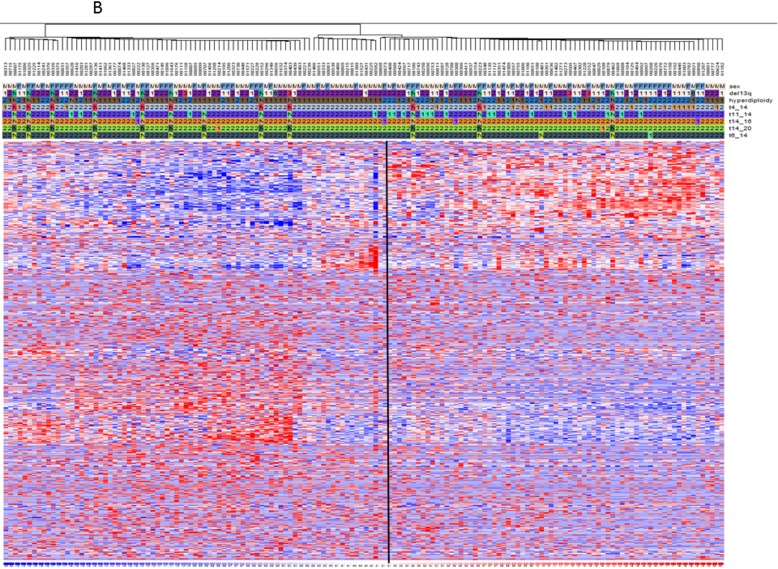
mRNA and miRNA signatures for hyperdiploid MM classification Heat maps showing the filtered mRNAs **A.** or miRNAs **B.** obtained by DChip Software. The genetic characteristics are reported including hyperdiploid status indicated as 1=HD-MM and 2=nHD-MM, N=not available and genetic alteration presence (1) or absence (2). Then standardized expression values (mean= 0, SD =1) for each molecule were analyzed through hierarchical clustering in DChip in order to show groups of mRNA and miRNA with similar expression changes. Clustering uses the Spearman correlation test between genes and samples and serves as the basis for merging nodes and building hierarchical trees. Finally clustered data were visualized through heat-maps. Colors represent respectively the down-regulation (scales of blue) and the up-regulation (scales of red). The standardized expression values most likely fall within [−2, 2]. By default, DChip uses pure white to represent 0, pure red to represent 2 or higher, and pure blue to represent −2 or lower.

**Figure 3:**
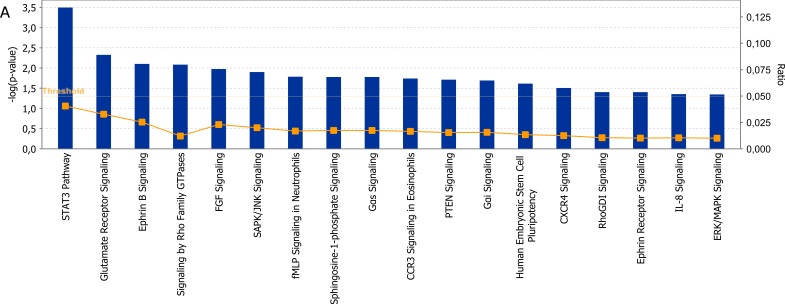

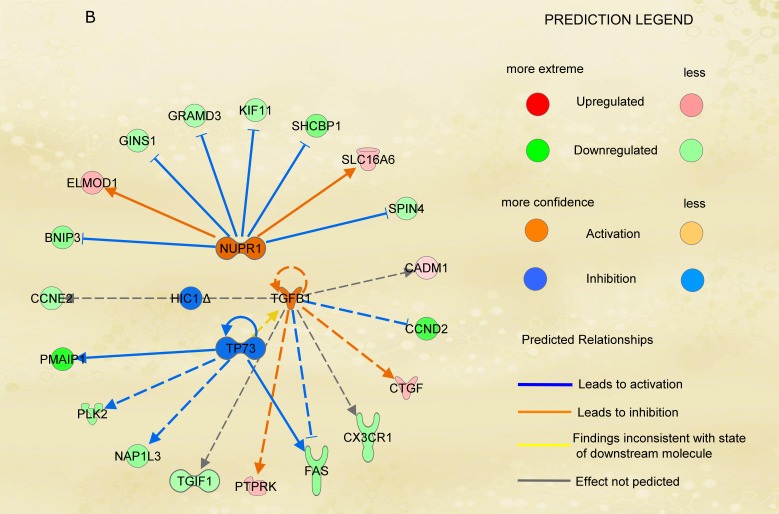
Functional analysis **A.** Top 20 signaling pathways identified by IPA include 39 selected molecules. The significance of association between selected genes and canonical pathway was evaluated by a scoring Fisher's Exact Test p-value (bars upper threshold line on y-axis). The ratio referring to the proportion of selected genes that matched the total number of molecules annotated in the related pathway (square dot line on the bar graph, bottom y-axis). The height of the bar represents the –log(p-value), thus is directly proportional to the statistical significance. **B.** Figure represents molecules involved in upstream regulator analysis integrated in a single networks. Figure shows upstream regulators (*NUPR1, TGFB1, TP73* and *HIC1*) their target genes as nodes of the network and relationships among them as edges. A gene is painted in red when it has been identified as up-regulated (i.e. over-expressed as revealed by fold-change analysis) while it painted in green when it is down-regulated. Each arrow links two genes whose the regulator mechanism is known. Arrows indicate both inhibition and activation through different colors. Yellow arrows indicate that the behavior of those genes in our dataset is divergent from what should be as predicted on a priori knowledge, and therefore needs additional investigation.

The clustering analysis was performed to elucidate the differences between the two classes on gene and miRNA expression. The heat-maps in Figure 2 and supplementary information indicate two main groups that include almost HD-MM in the right branch and the nHD-MM mainly clustered in the left branch, supporting our aim to further analyze these differences in deep. Consequently, we generated the lists of significantly differentially expressed (SDE)-genes and miRNAs in HD-MM. Our data are in line with previous evidence [15-18, 20], indicating that HD-MM are characterized by distinct transcriptional profiles, likely associated with the specific chromosomal alterations. Then, we annotated SDE-genes on the basis of known associated pathways and molecular functions, in order to discard uninformative transcripts. The IPA analysis identified the top 20 canonical pathways related to SDE-genes (Figure 3). Among the most modulated pathways, we found high perturbation of the Signal Transducer and Activator of Transcription (*STAT*)3 pathway (−Log(p-value)=3.5) together with other signaling pathways involved in cell proliferation and survival or intracellular signaling including *PTEN, IL-8* and *ERK/MAPK* signaling (Figure 3A). By canonical pathway analysis, we found among all the SDE-genes, a subset known as involved in some important pathways. Indeed we found that SDE-genes identified in previous step are related to *STAT3* pathways including genes involved in cell cycle progression (*Cyclin D1, D2*, and *c-Myc*), cell survival (*Bcl-xL, Bcl-2, Mcl-1*), angiogenesis (*HIF1α, VEGF*), cancer inflammation (NF*-KB, IL-6*). These findings confirm the initial hypothesis of a highly differentiated molecular scenario in HD-MM *versus* nHD-MM.

### *In silico* functional annotation

Functional annotations of SDE-miRNAs revealed main biological functions related to HD-MM *versus* nHD-MM. To better understand the hidden biology in complex ‘omics’ datasets, IPA provides insights into the molecular and chemical interactions and cellular phenotypes of the disease process. We found that the most significant functions that differentiated the two subtypes of MM patients were annotated as “cell growth and proliferation”, “cellular development” and “cell death” based on SDE-mRNAs (genes). Similarly, the same functional analysis performed on SDE-miRNAs revealed an overlapping scenario (supplementary information).

### Upstream regulator analysis

The IPA Knowledge base contains a large collection of experimental observations and findings obtained by integrating different data-sources including both biomedical literature and other databases. Consequently, IPA provides an underlying graph with more than 40.000 nodes (representing genes, proteins, miRNA, and other biologically active molecules and functions) and more than 1.400.000 edges representing different relations (e.g. causal relationships, or other effects related to expression, transcription, activation, molecular modification and transport). The knowledge base is used to perform the UR analysis (URA). In particular the algorithm embedded into IPA, determines likely URs (i.e. genes or molecules) that provide the regulation of the expression, analyzing the connections among such nodes and genes included in the dataset under investigation. The goal of URA is the understanding of regulatory mechanisms involved in data expression changes. In such a way URA is a powerful tool since the inference of URs allows the identification of potentially disrupted or altered mechanisms of regulators that may disclose potential druggable targets.

It must be underlined that URs do not necessarily represent TFs or other direct transcription regulators. URA tries to overcome the main limitations of classical approaches that are able to individuate SDE-genes and their related functions but lack in the individuation of causative relationship. By using URA, we may highlight causes of differential expression: *i*) TFs or TRs (upstream regulators, URs) known to modulate genes (identified as SDE-genes) based on Ingenuity embedded knowledge and *ii*) miRNAs experimentally identified as SDE-miRNAs in HD-MM *vs* nHD-MM patients that inhibit URs and their target genes. Moreover, we may also explain the effects of different modulation in terms of involved and downstream regulated targets.

Considering the overall scenario of Network Based Analysis, it should be noted that the main related approaches are MAGIA, mirConnX, dChipGemini, mirconnX and the previous Connectivity Map tool [32] that integrate mRNA and miRNA data and provide the analysis of regulatory networks.

With respect to those algorithms, it should be underlined that our approach has some advantages: i) IPA has a larger knowledge base that is periodically updated compared to the other approaches; ii) IPA offers the possibility to analyze even downstream effects, while other approaches analyze only regulatory mechanism among TF, miRNA and mRNA. Moreover, other tools such as PARADIGM, lack the possibility to explore the functional biology, considering that they offers the possibility to integrate only gene expression and copy number data.

Based on the differential gene expression data, URA identified 3 significant mechanisms. First, we identified the activation of *NUPR1* TF as candidate regulator of a defined set of down-regulated (*BNIP3*, *GINS1*, *GRAMD3*, *KIF11*, *SHCBP1 SPIN4)* or up-regulated *(ELMOD1* and *SLC16A6)* genes, according to our gene expression data (Figure 3B and Table S1). The other mechanism highlighted by URA integrates the regulators *TGFB1, TP73* and *HIC1*. As for *NUPR1, TGFB1* appears to be activated and different molecules related to this growth factor show expression as predicted by IPA analysis (Table S1). On the other hand*, HIC1* and *TP73* should be inhibited based on the down-regulation of the target molecules including *SPP1, CCNE2* and *FAS*. It is possible to appreciate that the activation or inhibition of the upper listed set of genes account properly in the our mRNA-SDE analysis. We also report an inconsistent finding among our experimental results and the prior knowledge about *TP73* and *TGFB1* relations. In fact, it is reported that *TP73* activate *TGFB1* expression [33], while in our dataset the inhibition of *TP73* is inconsistent with the activation of *TGFB1* clearly underlying the need of further investigations.

In order to better explain the functions carried out by these genes, we performed a functional analysis using as input only URs and related targets and we depict the enriched functions by overlapping these to the prior networks shown in supplementary information. In each figure, we show different enriched functions and we highlight corresponding molecules by evidencing them with a different border. Enriched functions are related to “cell growth” (Figure 2S-A), “cell cycle progression” (Figure 2S-B) and “tissue development” (Figure 2S-C). Since all these functions may be related to different cell progression behavior.

### miRNA-TR interplay

**Figure 4:**
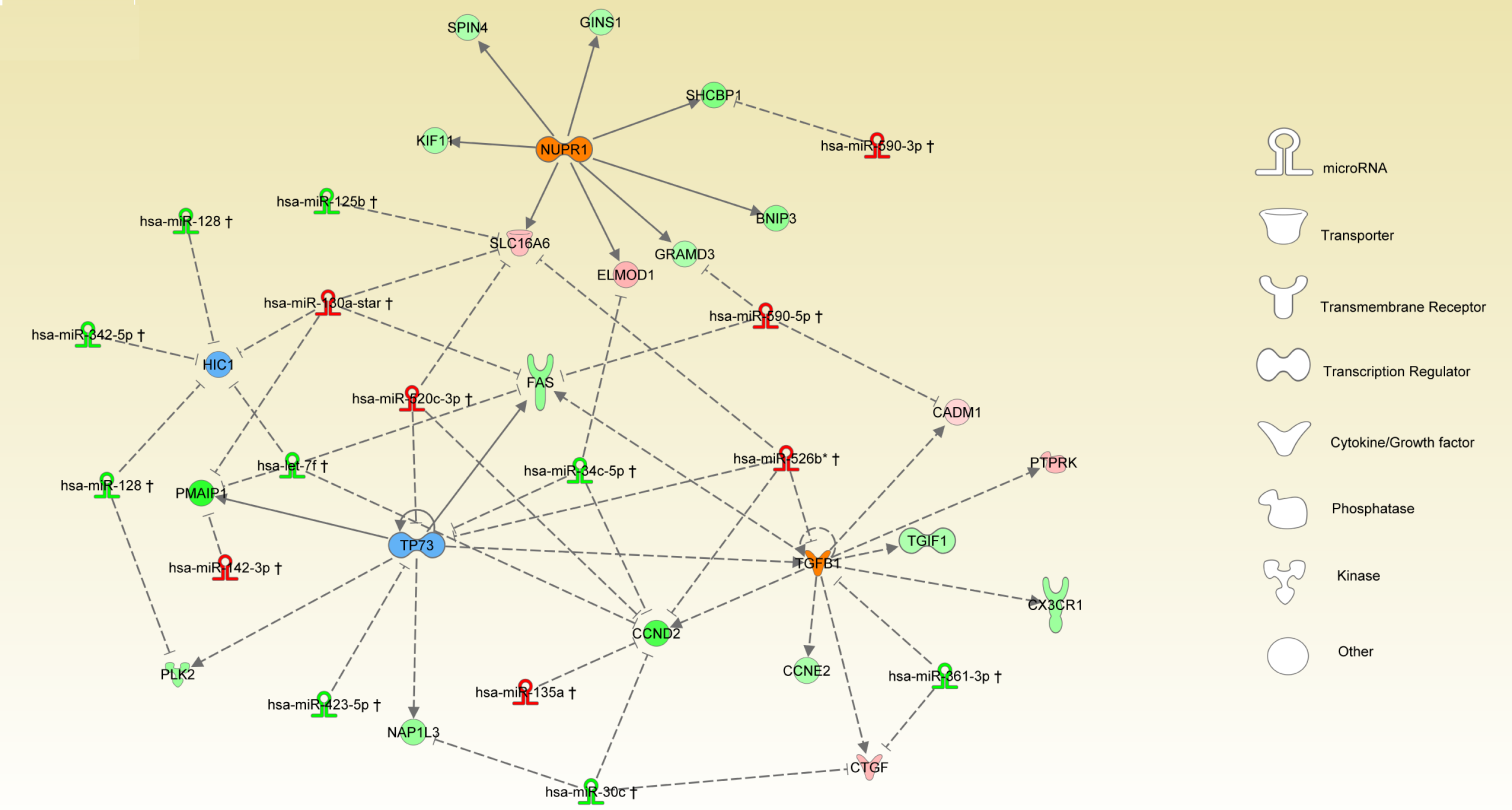
miRNA-TR interplay The network represents the integration of upstream regulator inference and miRNAs. Global regulatory network includes miRNAs and their targets selected between the top 4 URs and respective inferred genes. The nodes are URs with different shape for each function, colored orange or blue respectively for activation or inhibition. Circle are used for target signature genes. miRNAs and genes are colored in red or green as according for up- or down-regulation as reported in the SDE-genes and miRNAs lists. Connections between main players of the global regulatory network are depicted by lines where the relationships between miRNAs and target genes are shown as dashed lines with inhibitory function, while relationships between TRs and target genes are presented by lines either indicating activation or repression.

Integration of miRNA-target has been performed by IPA based on experimental and high confidence prediction using sources including Tarbase, TargetScan and IPA internal findings. In our method, we overlapped the prior knowledge obtained by URA analysis with experimentally observed data as deregulated miRNAs. The results of integration between SDE-miRNAs and targets, including inferred URs and relative targets detected as SDE-mRNA, are represented in Figure 4. Here, all relationships are in agreement with the model “miRNA inhibits targets”. In fact, considering the *NUPR1* inferred activation, two different down-regulated miRNAs were detected, miR-125b and miR-34c, targeting *SLC16A6* and *ELMOD1*, respectively. Both genes are up-regulated and may explain the activation of the *NUPR1*. On the other hand, miR-590-3p, which is up-regulated, may cause down-regulation of its target *GRAMD3* and similarly up-regulation of miR-590-5p may induce down-regulation of *SHCBP1*. On this last, it may exerts its inhibitory effect also the miR-520c-3p, that we detected as up-regulated in our model. Thus, the interplay miRNAs-URs-targets depicts a new regulatory scenario that differentially characterizes the 2 MM subgroups. In addition, over-expression of different miRNAs, including miR-130a and miR-590-5p, and the inferred activation of *TGFB1* may explain the down-regulation of *FAS*. Similarly, up-regulation of miR-520c-3p detected in the HD-MM may causes inhibition of *TP73* and *CCND2*. The latter are strongly down-regulated in HD-MM also for the inhibitory activity induced by over-expression of miR-520c-3p, miR-526b and miR-135a. Conversely, low expression of miR-30c and miR-361-3p in HD-MM may explain the up-regulation of *PTPRK* and *CTGF* genes. Finally, we observed that inhibition of *TP73* could be also affected by the over-expression of miR-142-3p and miR-130a and all together may induce a strong down-regulation of *PMAIP1* as detected in HD-MM cases.

**Figure 5:**
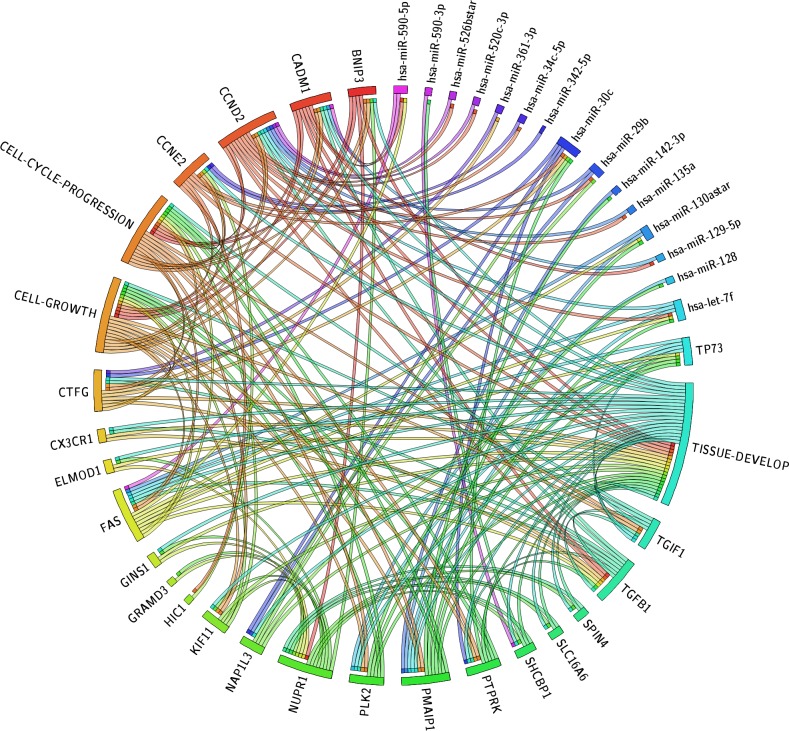
Circos plot The circos diagram visualize the dependency of the transcriptome represented in the form of top biological functions and of inferred upstream regulators: miRNAs, TRs and target mRNAs. The width of categories depends of the number of member molecules. In the circos plot we display these molecules with a segment and each connection with a ribbon. A connection between a TR and biological function means that this molecule was detected as activated or inhibited UR by upstream regulator analysis and that its target genes identified as SDE-genes grouping in the same functional category. A connection between a miRNA and a gene implies that this was the miRNA and its predicted target are regulated by UR. Finally, a connection between a gene and a biological function indicates it has annotation in the specific functional category. The Circos integrate all the relevant findings of the model we employed.

We have used Circos plot to integrate data and results obtained by IPA. We combined in a single graph mRNA, miRNA, URA and biological functions. In this way, we easily visualized the putative interactions of URAs and miRNAs on same target (Figure 5). We identified 9 miRNAs as most important post-transcriptional regulators of the URs. Most of them (5) direct regulate *TP73* and downstream effectors together with many genes (*FAS, TGIF1, PLK2, PMAIP1*) that in turn reflect their inhibitory effect on all described networks.

## DISCUSSION

The major aim of our work was to disclose the dynamic connection between the differential miRNome and transcriptome of the HD-MM *versus* nHD-MM. By a novel integrative genomic approach, we focused on the interplay of miRNAs, URs and target genes expression in the 2 subgroups on a comparative analysis. Recent reports highlight the relation between the genetic background of HD-MM or nHD-MM and the different clinical phenotypes, even if the genetic landscapes remain mostly undefined; nHD-MM is indeed defined on exclusion of HD and it is therefore a heterogeneous condition. Our experimental approach was an *in silico* functional analysis based on the transcriptomic (gene expression profiling) and the miRNomic background of HD-MM with nHD-MM patients. HD-MM patients showed significant gene and miRNA expression differences with nHD-MM patients based on heat maps reported (Figure 2 and supplementary materials), according to the knowledge that primary genetic events contribute to plasma cell immortalization and define the classification of MM. Prognostic subgroups have been defined using signatures derived from gene expression profiling [34, 35]. In this context the discovery of miRNA networks has revealed a new biological complexity. Also miRNA profiling has provided novel tools for disease stratification [16, 17, 36, 37]. However, these approaches do not capture all variability of clinical behaviors since they have been mainly designed for prognostic purposes and not for analysis of biologic complexity on multiple-level of interaction.

Based on the miRNA and gene expression data, 2 different branches are typically defined by hierarchical clustering profiles, identifying mostly HD-MM and nHD-MM subgroups. Gene expression by itself can identify HD-MM as recently described [20]. We here try to overcome the limitations of clustering-based approaches, which are able to define the differences between 2 classes in terms of different expression levels only, by the search of possible causes of these differences in terms of interplay of miRNAs and mRNAs. To this aim, we performed URA that enables a “system-level” point of view that may be used as first step for further analysis (e.g. the individuation of candidate targets for drugs). Classical gene expression analysis has demonstrated its effectiveness for diagnosis and prognosis of cancer by individuating different molecular subtypes with different clinical behavior. However, all these analyses lack on the individuation of causes, i.e. individuation of those genes that are directly related to cancer progression. The results of our integrated analysis suggest the possible effect of a subset of miRNAs and URs on a common set of targets and offer the opportunity of further orthogonal investigation of *TGFB1* and *TP73* interactions in HD-MM. In this view, the integrated analysis is the first step for the design and development of novel therapeutics based on information derived from integration and functional analysis of real-world data.

**Figure 6:**
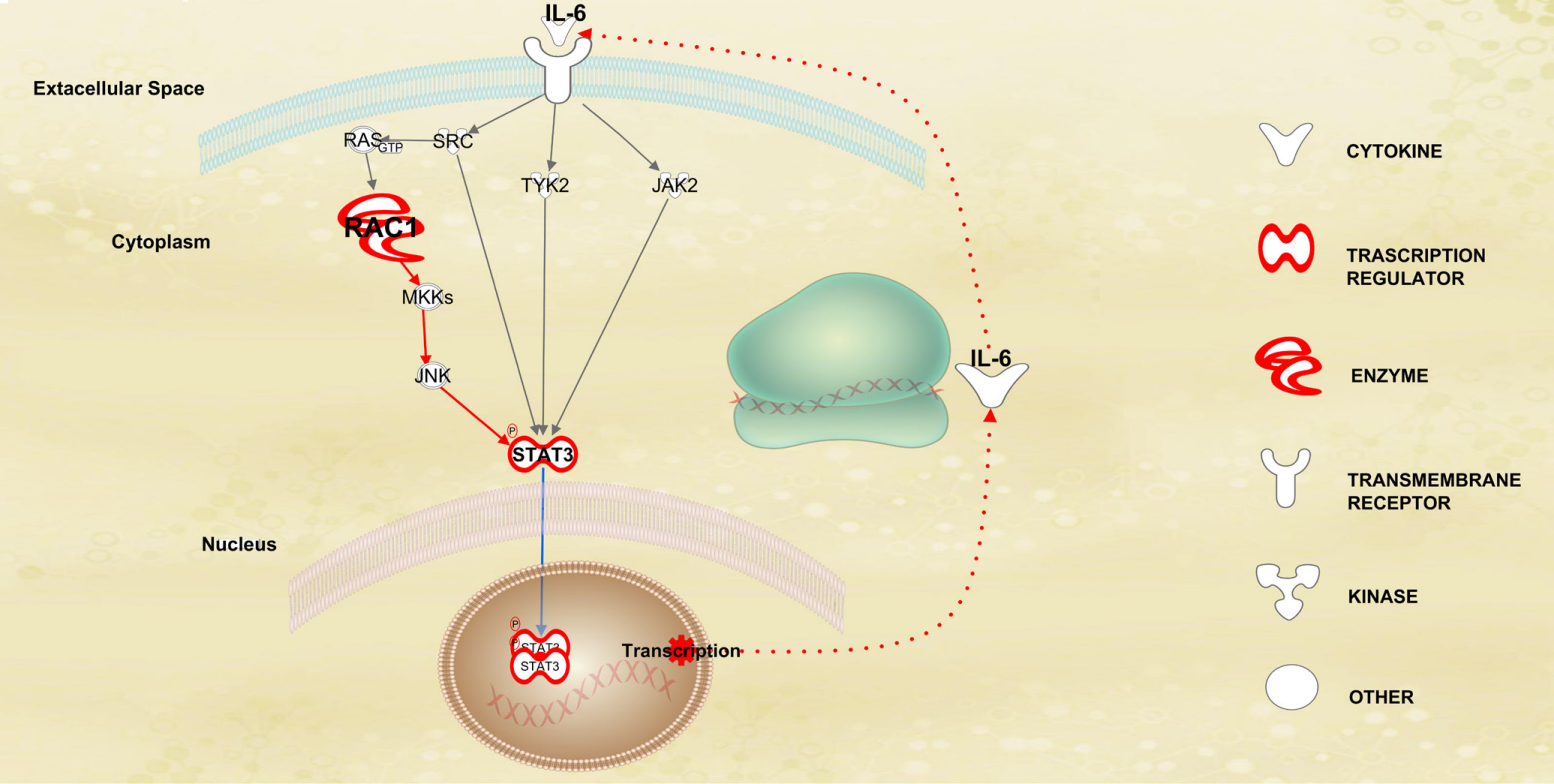
Dinamic features of STAT3 pathway in HD-MM Representation of the factors associated with the JAK-STAT pathway, detected by IPA analysis. The up-regulation of RAC1, a member of the Rho-GTPases family, induces STAT3 activation through an indirect feed-forward mechanism that involves the autocrine production and action of IL-6, a known mediator of STAT3, by the RAC1-mediated activation of MKK-JNK signaling. This circuitry establishes a link between oncogenic GTPase activity and Janus kinase-STAT signaling in HD-MM.

Among the top modulated canonical pathways in HD-MM, we first identified *STAT3* signaling, which is known to play an important role in MM-genesis through the regulation of growth and survival of MM cells. In particular, our analysis shows an up-regulation of *RAC1*, a member of the Rho-GTPases family, implicated in many cellular processes that influence cell proliferation, survival, motility and adhesion [38]. *RAC1* is able to induce *STAT3* activation through an indirect feed-forward mechanism that involves the autocrine production and action of IL-6 (Figure 6), a known mediator of *STAT3*, thus establishing a link between oncogenic GTPase activity and *Janus kinase-STAT* signaling [39]. Moreover, IPA upstream analysis revealed inhibition of *HIC1*, a tumor suppressor *p53*-activated gene located at chromosome 17p13.3, a region frequently deleted and epigenetically silenced in a variety of human cancers [40]. In addition to the DNA binding-mediated target gene regulation, *HIC1* could act via protein-protein interactions, inhibiting several cellular pathways including *JAK-STAT*. In fact, *HIC1* interacts with the DNA binding domain of *STAT3* attenuating *STAT3*-mediated transcription of several genes [41]. On this basis, the increased activity of *STAT3* signaling in HD-MM, may also rely on down-regulation of *HIC1* shown in these patients. Another important downstream target of *HIC1* is *SPP1*, also known as *Osteopontin* (*OPN*), which is found down-regulated by our analysis as a possible effect of the loss of activity of *HIC1*. *OPN* is a multifunctional bone matrix glycoprotein [42, 43], whose production in MM cells plays a critical role in bone disease by protecting the skeleton from destruction [44]. This finding indicates a potential cause of the described prevalence of bone disease in HD-MM patients which have low levels of *OPN*.

Among the TRs which result deregulated by IPA upstream analysis, the transforming growth factor (*TGFβ*) plays a key role in both HD-MM and nHD-MM pathogenesis. *TGFβ* family of growth factors controls a number of cellular responses and acts prominently in development and homeostasis of most human tissues. Disruption of the TGFβ pathway has been implicated in many human diseases, including solid and hematopoietic tumors. As a potent inhibitor of cell proliferation, *TGFβ* acts as a tumor suppressor; however in tumor cells, *TGFβ* loses anti-proliferative response and becomes an oncogenic factor [45]. In our analysis *TGFβ* is up-regulated in HD-MM, suggesting that in these patients it acts as an oncogene [46]. Moreover, hyper-activation of this pathway provides another potential mechanism of the frequency of bone disease in HD-MM group. In fact, *TGFβ* enhances proliferation of osteoblast progenitors and promotes mineralization of bone matrix, but at a later stage, *TGFβ* inhibits the late phases of differentiation of osteoblasts and represses mineralization of matrix [47]. Conversely, in nHD-MM patients, *TGFβ* acts mostly as tumor-suppressor because it's down-regulation is associated, in our model, with the up-regulation of *FAS* and *CCND2*. It is well known that *Fas*-mediated apoptosis plays an important role in activation-induced cell death, T-cell-induced cytotoxicity, immune privilege, tumor surveillance [48] and can also mediate a variety of non-apoptotic activities especially during tumor-genesis and tumor progression in *Fas*-resistant tumor cells [49]. In fact, *Fas* signaling is proposed to convert from a tumor suppressing to a tumor promoting activity, directly promoting apoptosis-resistant cancer cell growth and invasion [50]. Additionally, *Fas* signaling activation can induce secretion of pro-inflammatory cytokines and chemokines [51]. It was also described that Fas signaling promote lung cancer growth by recruiting myeloid-derived suppressor cells (MDSCs) *in vivo [52]* and the bioactive *Fas* ligand (*FasL*) released by activated T cells in exosomes promotes melanoma and lung cancer cell metastasis through *Fas* signaling. Therefore our data indicate that targeting *Fas* signaling and at the same time *Fas* signaling-initiated cancer-related inflammation may be helpful as a cancer therapeutic strategy in MM patients. This issues clearly needs to be further explored in follow-up studies. Although *Cyclins D* are the class of cell-cycle regulators more involved in MM pathogenesis, our analysis shows up-regulation of *Cyclin E2* which is related, with an effect not predicted by IPA, to downregulation of *HIC1*. Finally, *NUPR1*, a protein related to the high mobility group of TRs that is a key player in the cellular stress response and is involved in metastasis, is activated in HD-MM patients according to the target activation or inhibition. Among them *BNIP3* (down-regulated in HD-MM), a downstream target of *NUPR1*, is a novel hypoxia-inducible pro-apoptotic mitochondrial member of the *Bcl-2* family which acts as tumor-suppressor by regulating apoptosis in normal and malignant cells [53, 54]. At present the targets identified by our analysis are not exploited in the current treatment of MM.

In summary, our work confirms that miRNome and trascriptome of nHD-MM is different from HD-MM. These findings clearly support the hypothesis that HD-MM and nHD-MM do not differ only in early promoting events (mitotic crisis in HD-MM *versus* mostly translocations in nHD-MM), but evolve by different pathways which finally depict a highly divergent genetic architecture which is based on a branched Darwinian evolution [12]. Finally, our findings also suggest that several druggable targets are predominant in HD-MM and indicate new therapeutic strategies to be pursued in the next future. In particular, aberrant *STAT* signaling, as previously discussed, is recognized as a master regulator of tumor processes including proliferation, apoptosis, invasion, angiogenesis and cancer inflammation. Therefore, hyperactivity of *STAT3* in HD-MM suggests an important therapeutic target for several *STAT3* inhibitors, including peptide-mimetics, small molecule inhibitors and oligonucleotides, presently in pre-clinical and clinical development [55, 56]. Furthermore our analysis shows that *TGFβ* could be considered as a therapeutic target to disrupt the pathognomonic skewed cellular interactions in MM bone marrow microenvironment in the aim to antagonize bone destruction and MM-related bone disease. All these findings on miRNA interactions with mRNAs and URs, might acquire additional importance taking into account their relevance as therapeutic targets/agents as recently reported in MM [57-73]. Integrative genomics may offer a reliable powerful tool to select molecular targets for therapeutic interventions.

For further multiple level analysis, the integration of RNA expression data and proteomic profiling and/or DNA methylation data, may drive to the identification of pathways predictive of favorable response and microenvironment interactions with clinical relevance in MM treatment [74, 75]. In addition, the integration of genomic sequencing and mutational profiling may provide the genomic landscape underlying MM development and indicates potential druggable targets in this malignancy. This approach is likely to outperform single level analyses as for instance Next Generation Sequencing (NGS)-based personalized therapeutic strategies. Although NGS is capable to identify druggable mutations in the majority of human cancers [76]. It is important to consider that driver actionable targets needs to be identified taking into account the general biologic scenario which can be disclosed only by multiple level integrated analysis.

On the basis of our findings, HD-MM should be considered in clinical trial stratification. Potential targeted approaches should be therefore investigated for HD-MM, for instance taking into account the nodal points that emerge from our integrated analysis. Moreover, we suggest that follow-up studies should investigate the prognostic/predictive value of the inferred targets in patient series stratified for HD molecular phenotype.

In conclusion, our study indicates that an integrative genomics approach can identify key URs based on expression profiles and on external knowledge. Functional studies can be now designed to provide the final wet biology proof of our *in silico* findings. It must be considered that the main limitation of integrative genomics is its inherent hypothesis generating aim. A strongly defined path from integrative genomics to well defined proof-of-concept orthogonal studies appears a provocative novel translational approach in MM, whose cure is a still unmet goal.

## MATERIALS AND METHODS

### Patients and miRNA and mRNA microarray data

To investigate the dynamic regulation and potential co-regulation of mRNAs and miRNAs, we used the MM miRNAs and gene expression microarray data obtained by the Gene Expression Omnibus (GEO). Gene expression profiling (GEP) were performed using the Affymetrix HG-133 Plus 2.0 Genechip arrays (accession number GSE15695) while miRNA profiles were generated by Affymetrix GeneChip miRNA arrays v1.0 (accession number GSE41276). We included in this study a total of 152 patients. Among them, 139 had available GEP, miRNA profiles and hyperdiploid status assessed by FISH (HD=83; nHD=56).

### Data analysis

The workflow of the data processing and analysis is illustrated in Figure 1. Pre-processing of microarray data was performed by Affymetrix^®^ Expression Console^TM^ software using the RMA normalization algorithm for gene expression data and Affymetrix^®^ QCTool for miRNA data. Then to remove the number of uninformative features the mRNA and miRNA expression data were filtered with options for logged data, by DChip^®^ software obtaining a list of 517 annotated transcript and 708 hsa-miRs, respectively. Hierarchical clustering analysis, using correlation as distance metric and centroid linkage, of the filtered genes/miRNAs was performed by DChip and the representation by heat map are shown in Figure 2 and in supplementary materials. To identify significant differentially expressed (SDE) genes and miRNAs we performed comparison analysis by DChip software using 139 MM patients where the hyperdiploid status were known. We searched for SDE genes/miRNAs using the two groups HD-MM (Experiment -E) *versus* nHD-MM (Baseline -B). The fold change (FC) was calculated as E-B (>+1,2) for genes and E/B (>+1,2) for miRNAs. We obtained a list of 39 differential expressed mRNA (SDE-genes) and 343 miRNAs (SDE-miRNAs), respectively.

#### Ingenuity pathway analysis

Both SDE-mRNAs and SDE-miRNAs between HD-*versus* nHD-MM lists were uploaded on the IPA software (www.ingenuity.com, License Item Number IPA-NUL-00001) to analyze first canonical pathways linked to the two distinct classes of myeloma, based on the IPA library of canonical pathways signaling. The statistical significance of the association among the experimental conditions and each discovered pathway was measured by Fisher's exact Test. Consequently, we discarded those pathways with P-value higher than 0.05, ensuring that the association between the genes in our data set and a canonical pathway was not explained by chance alone.

#### IPA functional analysis

To identify significant biological functions in our gene expression data set, we used the list of SDE genes and selected only biofunctions with a P-value lower than 0.05 using the Benjamini–Hochberg correction.

#### miRNA target filter

IPA stores association among miRNAs and their targets by integrating different sources: Tarbase, TargetScan and its internal findings. We uploaded SDE-miRNAs list into IPA and then we performed miRNA target filtering. In order to improve the quality of results, we chose only experimental and high confidence predicted targets.

#### Upstream regulator analysis

The Upstream Regulator Analysis (URA) is based on a statistical matching that determines and ranks regulators that may be associated to the dataset [31]. The approach evaluates the relationships by using two measures: an overlap P-value that measures the statistical significance of the finding, as well as an activation Z-score that carries information about the strength of the relationship and the activation state (either activated or inhibited). Z-score varies in the range −2 (inhibition) and +2 (activation). In our data IPA upstream regulators analysis was performed using the SDE gene list. Results were sorted by statistical and biological significance using both P-value and Z-score. We selected the upstream regulators (URs) with P-value higher than 0,05 and Z-score +1,9. Then we performed by IPA the overlay of regulatory networks and bio-function.

#### miRNA-TR interplay

For each molecule individuated in the previous step, we searched for related miRNAs into the SDE-miRNA list. In order to improve the quality of findings we selected only miRNAs identified as experimental or high score predicted for IPA. Thus, we were able to elucidate interplay of upstream and miRNA regulators of the same target.

### Circos plot

We used Circos Plot to better visualize the correspondence among identified SDE-mRNAs, the related miRNA and their bio-functions [77]. Thus we linked each SDE-mRNA to their related miRNA and URs, and to the function assessed.

## SUPPLEMENTARY FIGURES AND TABLES


