# Supplementary material for: Integrated analysis of microRNAs, transcription factors and target genes expression discloses a specific molecular architecture of hyperdiploid multiple myeloma
Source: Oncotarget. 2015 May 27;6(22):19132–47. doi: 10.18632/oncotarget.4302 (PMC4662480; doi:10.18632/oncotarget.4302)
Supplement: Supplementary file 1 [file oncotarget-06-19132-s001.pdf]

# Integrated analysis of microRNAs, transcription factors and target genes expression discloses a specific molecular architecture of hyperdiploid multiple myeloma

**Supplementary Material**

Fig. 1S A

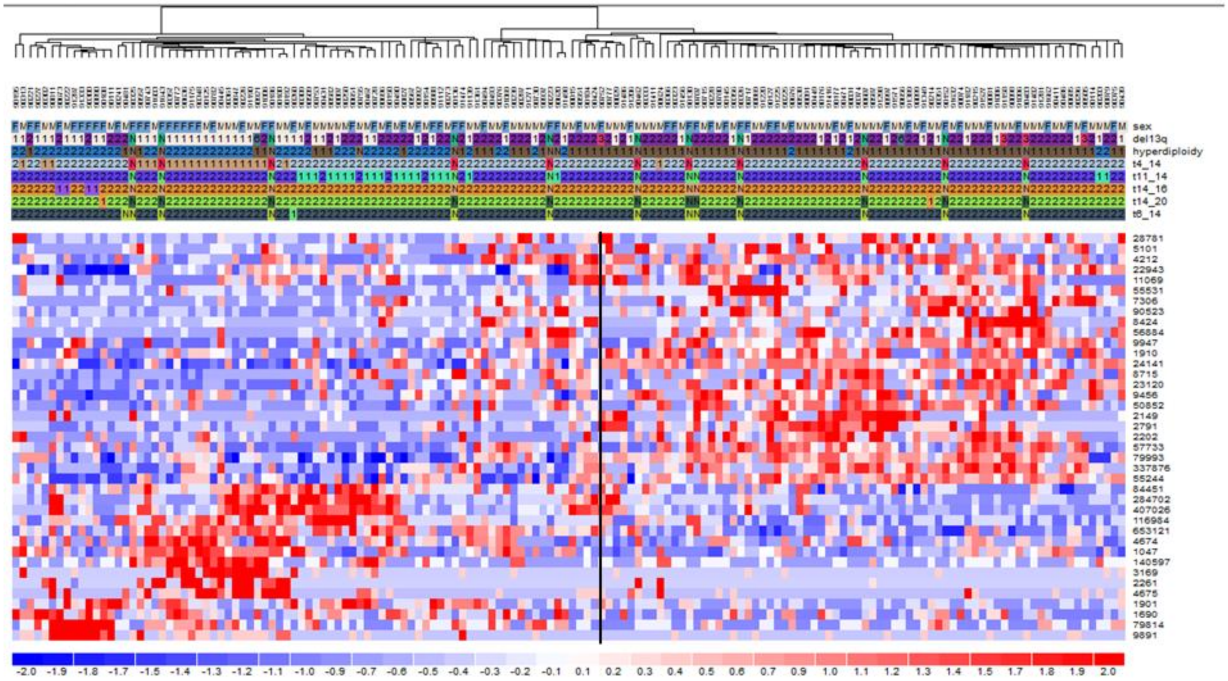

Fig. 1SB

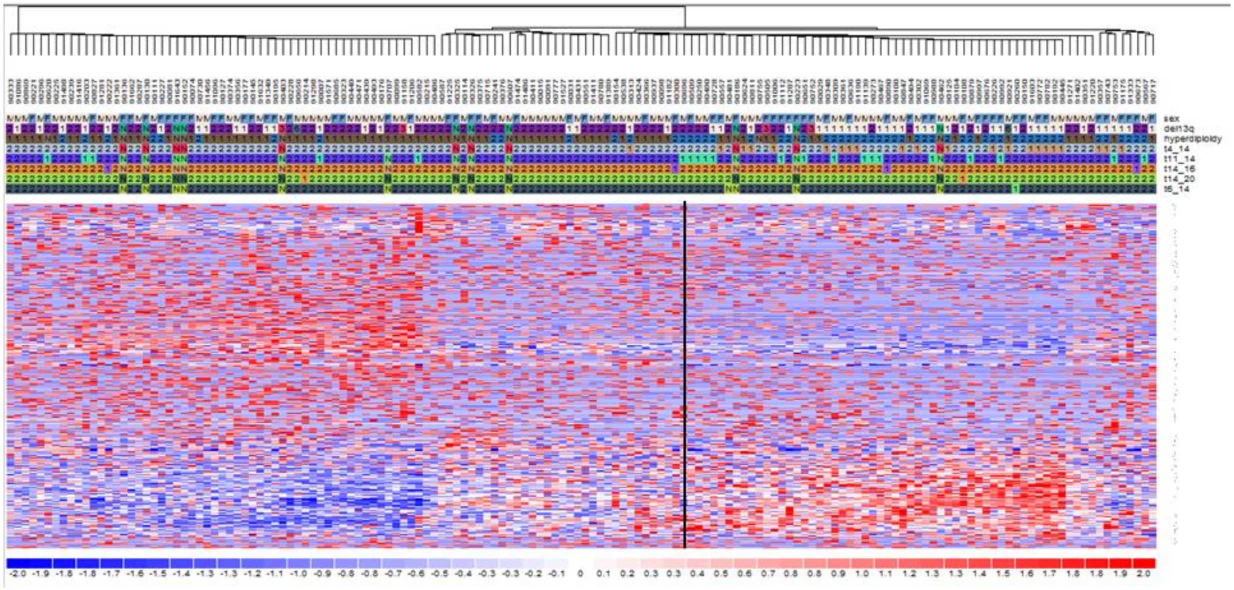


**Figure 1S: Significant differential expressed genes and microRNA signatures for hyperdiploid MM classification.**

Heat maps showing the significant differential expressed mRNAs (39) or miRNAs (343) detected by using DChip Software. The genetics characteristics are reported including hyperdiploid status indicated as 1=HD-MM and 2=nHD-MM, N=not available and genetic alteration presence (1) or absence (2). Then standardized expression values (mean= 0, SD =1) for each molecule were analyzed through hierarchical clustering in DChip in order to show groups of mRNA and miRNA with similar expression changes. Clustering uses the Spearman correlation between genes and samples and serve as the basis for merging nodes and building hierarchical trees. Finally clustered data were visualized through heatmaps. Colors represent respectively the down-regulation (scales of blue) and the up-regolation (scales of red). The standardized expression values most likely fall within [-2, 2]. By default, DChip uses pure white to represent 0, pure red to represent 2 or higher, and pure blue to represent –2 or lower.

A)The top 39 significantly expressed and annotated mRNAs fall mainly into two main groups. Affymetrix probe_set ID were reported

B)The 343 SDE-miRNAs reflects the mRNAs profile and cluster in the two subtype MM groups.

Fig.2S

**A. Overlay with Cell Growth**

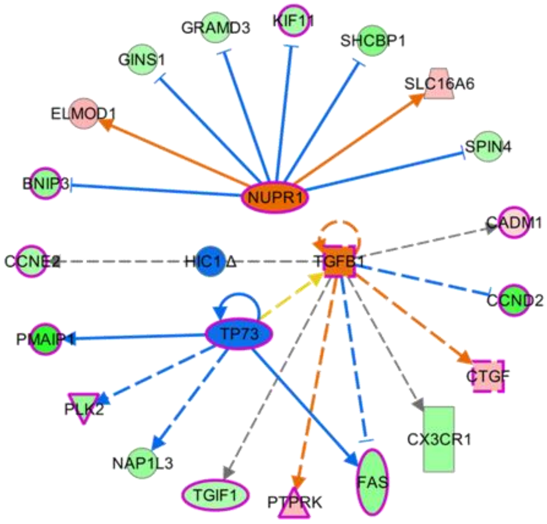


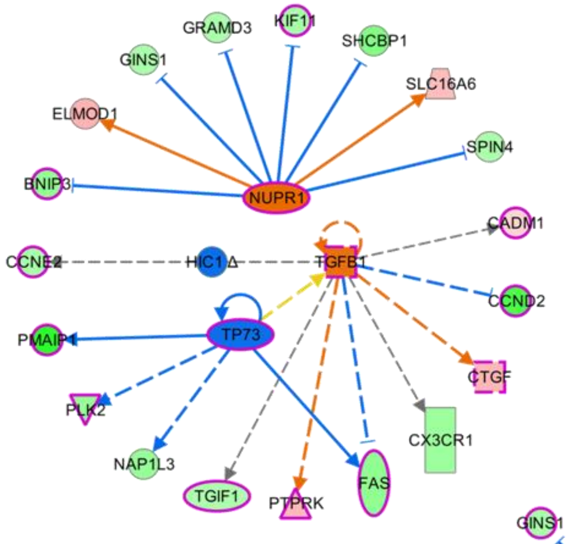


**B. Overlay with Cell Cycle Progression**

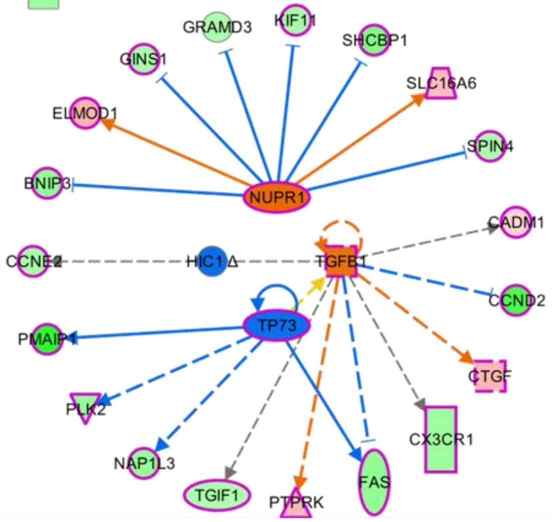


**C. Overlay with Tissue Development**

**Figure 2S: Overlay of regulatory Networks and Biofunction**.

This picture shows (evidenced with pink-painted borders) the overlay of bio-functions and of modulated genes involved in URA. This shows how the different regulation of this molecules may be related to different cell progression behavior and finally may explain the different progression of tumor in two groups. In each figure we represent a different enriched functions and we highlight corresponding molecules by evidencing them with a different border. Enriched functions are related to cell growth (A), cell cycle progression (B) and tissue development (C).

## TABLE 1. Target molecules in the dataset

INHIBITED

HIC1 ← INHIBITED → TP73

| Genes in dataset | Prediction (based on expression direction) | Other | Findings | Genes in dataset | Prediction (based on expression direction) | Other | Findings |
|---|---|---|---|---|---|---|---|
| **SPP1** | **Inhibited** | **-0,71** | **Upregulates** | **PLK2** | **Inhibited** | **-0,65** | **Upregulates** |
| **CCNE2** | **Inhibited** | **-0,54** | **Upregulates** | **NAP1L3** | **Inhibited** | **-0,66** | **Upregulates** |
| **CA2** | **Inhibited** | **-0,67** | **Upregulates** | **FAS** | **Inhibited** | **-0,69** | **Upregulates** |
| **AMIGO2** | **Inhibited** | **1,16** | **Downregulates** | **PMAIP1** | **Inhibited** | **-1,33** | **Downregulates** |

ACTIVATED

TGFB1 ← ACTIVATED → NUPR1

| Genes in dataset | Prediction (based on expression direction) | Other | Findings | Genes in dataset | Prediction (based on expression direction) | Other | Findings |
|---|---|---|---|---|---|---|---|
| **CTGF** | **Activated** | **0,86** | **Upregulates** | **SPIN4** | **Activated** | **-0,520** | **Downregulates** |
| **PTPRK** | **Activated** | **0,84** | **Upregulates** | **SLC16A6** | **Activated** | **0,780** | **Upregulates** |
| **CADM1** | **Affected** | **0,51** | **Regulates** | **SHCBP1** | **Activated** | **-0,790** | **Downregulates** |
| **TGIF1** | **Affected** | **-0,51** | **Regulates** | **KIF11** | **Activated** | **-0,550** | **Downregulates** |
| **CCNE2** | **Affected** | **-0,54** | **Regulates** | **GRAMD3** | **Activated** | **-0,520** | **Downregulates** |
| **CX3CR1** | **Affected** | **-0,62** | **Regulates** | **GINS1** | **Activated** | **-0,540** | **Downregulates** |
| **FAS** | **Activated** | **-0,69** | **Downregulates** | **ELMOD1** | **Activated** | **0,920** | **Upregulates** |
| **CCND2** | **Activated** | **-1,13** | **Downregulates** | **BNIP3** | **Activated** | **-0,690** | **Downregulates** |

In the table are reported the URAs results as inhibited or activated UR molecules. The downstream genes identified in the SDE-genes are reported with relative FC value. Findings assigned by IPA are reported as upregulated or downregulated according to IPA annotation, where regulated indicate the both activity, up and down described functions
